# Linear Stability of a Viscoelastic Liquid Film on an Oscillating Plane

**DOI:** 10.3390/nano15080610

**Published:** 2025-04-16

**Authors:** Jing Zhang, Quansheng Liu, Ruigang Zhang, Zhaodong Ding

**Affiliations:** School of Mathematical Science, Inner Mongolia University, Hohhot 010021, China; 18648067752@163.com (J.Z.); smslqs@imu.edu.cn (Q.L.); rgzhang@imu.edu.cn (R.Z.)

**Keywords:** Oldroyd-B fluid, liquid film, linear stability, Floquet system, long-wave mode

## Abstract

This paper investigates the linear stability of the liquid film of Oldroyd-B fluid on an oscillating plate. The time-dependent Orr–Sommerfeld boundary-value problem is formulated through the assumption of a normal modal solution and the introduction of the stream function, which is further transformed into the Floquet system. A long-wavelength expansion analysis is performed to derive the analytical solution of the Orr–Sommerfeld equation. The results indicate that long-wave instability occurs only within specific bandwidths related to the Ohnesorge number (Oh). Fixing the elasticity parameter (El) and increasing the relaxation-to-delay time ratio ($\tilde{\lambda }$) from 2 to 4 or fixing ($\tilde{\lambda }$) and increasing (El) from 0.001 to 0.01 decreases the number of unstable bandwidths while enhancing the intensity of unstable modes. Increasing the surface-tension-related parameter ($\zeta^{*}$) from 0 to 100 suppresses the wave growth rate, stabilizing the system. Additionally, increasing ($\tilde{\lambda }$) from 2 to 4 reduces the maximum values of the coupling of viscoelastic, gravitational, and surface-tension forces, as well as the maximum value of the Floquet exponent, further stabilizing the system. These findings provide supplements to the theoretical research on the stability of viscoelastic fluids and also offer a scientific basis for engineering applications in multiple fields.

## 1. Introduction

The study of non-Newtonian fluids remains a dynamic and critical field in contemporary technological development and industrial advancement. Particularly, the exploration and application of viscoelastic fluids have garnered significant research interest in recent decades due to their drag-reducing properties in industrial processes [1]. Furthermore, owing to their unique mechanical characteristics, such fluids are widely employed in coating, crystal growth, and material processing [2]. On the other hand, instabilities in viscoelastic liquids arise in diverse applications, including lubrication, coating, and polymer processing operations [3,4].

Currently, in many engineering problems, the stability of thin-film flows near walls is critically important. However, instabilities often arise and cannot be neglected. Related research topics, such as the study of the stability of free-surface flows on oscillating planes, hold even greater significance. These studies have diverse applications in atomization technologies, including fuel spray formation, high-tech surface cleaning, and advanced material processing [5,6]. Unlike the stability of base flows, problems involving time-dependent base flows are challenging to handle even numerically. For the flow of Newtonian fluids on horizontally oscillating planes, Yih [7] first theoretically investigated the stability of a single-layer flow with a freely deformable upper surface on an oscillating plate. Using a long-wave expansion and Floquet theory, he solved the time-dependent Orr–Sommerfeld boundary value problem. Yih found that in the absence of gravity, this long-wave mode instability was independent of the oscillation amplitude of the plate. As the oscillation frequency increased, alternating regions of stability and instability emerged. When gravity was considered, instabilities arose within regions corresponding to specific bandwidths of the applied frequency, provided the modulation amplitude was sufficiently large. The critical Reynolds number increased rapidly with higher oscillation frequencies. The results indicated that flow stability depends on the Froude number and oscillation frequency, with long-wavelength instabilities existing only within certain separated frequency bandwidths. Or [8] extended this analysis to investigate the same problem for arbitrary wavenumbers and discovered that finite-wavelength instabilities occur once the imposed frequency exceeds a certain threshold. The results revealed that the neutral stability curve for long-wave instability is U-shaped, and through branch points detected on the long-wave neutral curve, a set of monotonic neutral curves associated with finite-wavelength instabilities emerges. In practice, due to the competition between long-wave and finite-wavelength modes, the finite-wavelength modes become more unstable than the long-wave modes over specific ranges of the applied frequency. Or and Kelly [9] examined the effects of wall oscillations and thermocapillary phenomena on the instability of fluid layers. They found that for long-wavelength thermocapillary convection, oscillatory shear could either stabilize or destabilize the base state, depending primarily on the imposed forcing frequency. Under microgravity conditions, the significant stabilization of dominant long-wavelength convection could be achieved by properly selecting the applied frequency. Burya and Shkadov [10] studied the stability of a liquid film flow along an inclined plate under gravity with periodic oscillations, incorporating the effects of surface tension. They compared the stability of disturbed flows and steady flows at small and large inclination angles. The research found that, in the first case (small inclination), oscillations could amplify Tollmien–Schlichting waves, while the generated resonant waves exhibited instability across a range of wavenumbers. In the second case (large inclination), long surface waves on the nearly vertical plate were observed to be stable; however, unstable resonant waves developed within specific parameter ranges. As the inclination angle of the plate decreased, the surface waves ceased to remain stable.

Subsequently, Dandapat and Gupta [11] extended this research to study viscoelastic liquids on oscillating plates, conducting a linear stability analysis in the long-wave regime. They discovered that the influence of viscoelastic parameters on long-wave modes exhibited frequency dependence: destabilizing effects occurred within specific frequency bands, while stabilizing effects dominated in others, though finite-wavelength modes were not considered. Building on these conclusions, Samanta [12] explored the stability of infinitesimal perturbations with arbitrary wavenumbers. He observed that long-wave instability regions emerged within separated bandwidths of the applied frequency. Outside these frequency bands, long-wave disturbances remained stable. In fact, the stabilizing role of viscoelasticity on long-wave modes was most pronounced within these stable frequency bands. However, for finite wavenumbers, no stable frequency bands existed, as finite-wavenumber modes appeared within these “stable” bands predicted by long-wavelength analysis. Notably, in the presence of viscoelastic parameters, the most unstable modes exhibited greater instability compared to those in Newtonian liquids.

As a canonical viscoelastic model, the Oldroyd-B fluid [13] has attracted considerable attention due to its intrinsic coupling of viscous dissipation and elastic energy storage. Recent studies include Fu et al.’s [14] analysis of Kelvin–Helmholtz instability in annular Oldroyd-B fluid films with heat and mass transfer in gas-confined pipes and Ahmad et al.’s [15] investigation of magnetohydrodynamic thin-film flows, which revealed velocity field suppression with the increase in magnetic field strength, film thickness, delay time, and relaxation time parameters.

While these studies have significantly advanced our understanding of Oldroyd-B fluid dynamics under various conditions, critical knowledge gaps persist regarding the stability mechanisms of such fluids on oscillatory substrates. In this context, this study fills the above research gap by investigating the linear stability of Oldroyd-B fluid film on an oscillating flat surface. We derive the time-containing Orr–Sommerfeld equation, construct the Floquet system, and systematically analyze the combined effects of the elastic parameters El, relaxation–delay time ratio $\tilde{\lambda }$, surface-tension-related parameter $\zeta^{*}$, and the Ohnesorge number Oh on the flow stability. Based on the long-wave approximation and Floquet theory, it is found that long-wave instability occurs only within the bandwidth associated with a specific Oh; increasing the elasticity parameter El or the relaxation–delay time ratio $\tilde{\lambda }$ reduces the number of unstable bandwidths, and the surface tension stabilizes the system by decreasing the long-wave modal growth rate. These findings deepen the theoretical knowledge of the stability of non-Newtonian fluids under oscillatory conditions.

## 2. Mathematical Formulation

This study focuses on the flow characteristics of an incompressible two-dimensional viscoelastic liquid film on an oscillating horizontal plane. The viscoelastic liquid film has a density of ρ, a viscosity of μ, and a surface tension of σ. The liquid film starts to move due to the vibration of a flat plate with a basic velocity of $\hat{i}U_{0}\cos\omega t$ in the horizontal direction, where $U_{0}$ is the amplitude, ω is the frequency, and $\hat{i}$ is the unit vector in the flow direction.

**Figure 1:**
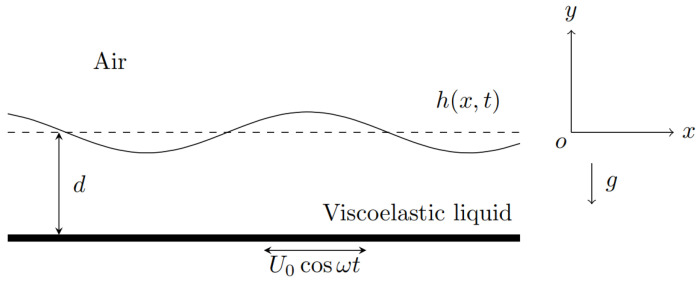
Model diagram of liquid film flow.

Figure 1 shows the model of this flow (taking into account the influence of gravity). The origin of the coordinate system is located at the free surface of the liquid film [8,12]. In fact, selecting the coordinate origin at the free surface of the liquid film or at the solid plate yields identical computational results, making these two choices mathematically equivalent. Therefore, we directly adopted the coordinate system described in the referenced literature. Since free-surface deformations are typically small-amplitude perturbations, placing the origin at the free surface allows us to assume the interfacial perturbation takes the form $h=\tilde{h}$ (where $\tilde{h}$ denotes a small disturbance). The *x*-axis is along the flow direction, and the *y*-axis is perpendicular to the flow direction. The velocity components in the flow direction and the perpendicular direction are denoted as $u_{x}=u$ and $u_{y}=v$, respectively. The gravity of the liquid film cannot be ignored, and the direction of the gravitational acceleration is vertically downward, with the thickness of the liquid film being *d*. The thickness d of the liquid film is a crucial parameter in this model. In the subsequent work, it serves as the characteristic scale of length, directly determining multiple important dimensionless parameters and governing the main physical mechanisms of the flow.

### 2.1. Governing Equations

The continuity equation and momentum equation that govern the flow of the liquid film are

(1) $$\partial_{i}u_{i}=0,$$

(2) $$\rho \left(\partial_{t}u_{i}+u_{j}\partial_{j}u_{i}\right)=\rho g_{i}+\partial_{j}T_{ij}.$$

In Equation (2), $T_{ij}$ represents the stress tensor. The subscripts *i* and *j* are summed over the *x* and *y* directions, respectively. Specifically,

(3) $$g_{x}=0,\;g_{y}=-g,\;T_{ij}=-pI_{ij}+\tau_{ij}.$$

where *g* is the magnitude of the gravitational acceleration, *p* is the isotropic pressure, $I_{ij}$ is the Kronecker symbol, and $\tau_{ij}$ is the deviatoric stress tensor. The definition of the deviatoric stress tensor $\tau_{ij}$ is determined by the constitutive equation of the Oldroyd-B fluid, and its specific form is shown in the following equation:

(4) $$\tau_{ij}+\lambda_{1}\overset{\nabla }{\tau_{ij}}=\mu \left({\dot{\gamma }}_{ij}+\lambda_{2}\overset{\nabla }{{\dot{\gamma }}_{ij}}\right).$$

The Oldroyd-B fluid model considered in this paper defines the behavior of a viscoelastic fluid through the above-mentioned specific constitutive equation. Here, μ is the viscosity coefficient, ${\dot{\gamma }}_{ij}$ is the rate-of-deformation tensor, its expression form is as follows:

(5) $${\dot{\gamma }}_{ij}=\frac{1}{2}\left(\frac{\partial u_{i}}{\partial x_{j}}+\frac{\partial u_{j}}{\partial x_{i}}\right)$$

$\lambda_{1}$ and $\lambda_{2}$ are the stress relaxation time and the deformation delay time, respectively. The limiting case where $\lambda_{1}=\lambda_{2}=0$ corresponds to a purely viscous (Newtonian) fluid. And the corotational derivative of $\tau_{ij}$ is defined as:

(6) $$\overset{\nabla }{\tau_{ij}}=\frac{\partial \tau_{ij}}{\partial t}+u_{k}\frac{\partial \tau_{ij}}{\partial x_{k}}-\tau_{kj}\frac{\partial u_{i}}{\partial x_{k}}-\tau_{ik}\frac{\partial u_{j}}{\partial x_{k}}.$$

The corresponding boundary conditions are as follows:

(i) Boundary conditions at the flat plate: at y=−d
  (7) $$u_{x}=u=U_{0}\cos\omega t,\;u_{y}=v=0.$$
(ii) Dynamic boundary conditions at the free surface of the liquid film (including those in the tangential and normal directions): at y=h(x,t)
  (8) $$\tau_{ij}n_{j}t_{i}=0,$$
  (9) $$\tau_{ij}n_{i}n_{j}=\frac{\sigma \partial_{xx}h}{\sqrt{{\left[1+{\left(\partial_{x}h\right)}^{2}\right]}^{3}}}.$$
  where $n=(n_{x}\hat{i}+n_{y}\hat{j})$ is the unit normal vector, $t=(t_{x}\hat{i}+t_{y}\hat{j})$ is the unit tangent vector, and $\hat{j}$ is a unit vector in the cross-streamwise direction.
(iii) Kinematic boundary conditions at the free surface of the liquid film:
  (10) $$\partial_{t}f+u_{i}\partial_{i}f=0.$$
  where f(x,t)=h(x,t)−y.

Next, we transform the governing Equations (1) and (2) and the constitutive Equation (4) into their component forms.

(11) $$\frac{\partial u}{\partial x}+\frac{\partial v}{\partial y}=0,$$

(12) $$\rho \left(\frac{\partial u}{\partial t}+u\frac{\partial u}{\partial x}+v\frac{\partial u}{\partial y}\right)=-\frac{\partial p}{\partial x}+\frac{\partial \tau_{xx}}{\partial x}+\frac{\partial \tau_{xy}}{\partial y},$$

(13) $$\rho \left(\frac{\partial v}{\partial t}+u\frac{\partial v}{\partial x}+v\frac{\partial v}{\partial y}\right)=-\rho g+\frac{\partial \tau_{yx}}{\partial y}-\frac{\partial p}{\partial y}+\frac{\partial \tau_{yy}}{\partial y},$$

(14) $$\tau_{xx}+\lambda_{1}\left(\frac{\partial \tau_{xx}}{\partial t}-2\tau_{yx}\frac{\partial u}{\partial y}\right)=-2\mu \lambda_{2}{\left(\frac{\partial u}{\partial y}\right)}^{2},$$

(15) $$\tau_{yy}+\lambda_{1}\frac{\partial \tau_{yy}}{\partial t}=0,$$

(16) $$\tau_{xy}+\lambda_{1}\left(\frac{\partial \tau_{yx}}{\partial t}-\tau_{yy}\frac{\partial u}{\partial y}\right)=\mu \frac{\partial u}{\partial y}+\mu \lambda_{2}\frac{\partial^{2}u}{\partial t\partial y}.$$

From Equation (15), it can be obtained that

(17) $$\tau_{yy}=B\left(y\right)e^{-\frac{t}{\lambda_{1}}}.$$

where B(y) is an arbitrary function. When t<0, $\tau_{yy}\to 0$. From this, we can conclude that B(y) must be 0, and thus we obtain $\tau_{yy}=0$. According to the reference [16,17], if the fluid is at rest at t=0, then τ(y,0)=0. Based on this, when t<0, τ(y,t)=0, which also means that when t<0, $\tau_{yy}$ also approaches 0. Therefore, B(y) must be 0.

Substitute $\tau_{yy}=0$ into Equation (16), and we get

(18) $$\tau_{xy}+\lambda_{1}\frac{\partial \tau_{yx}}{\partial t}=\mu \frac{\partial u}{\partial y}+\mu \lambda_{2}\frac{\partial^{2}u}{\partial t\partial y}.$$

After combining Equations (12) and (13) with Equation (11) and taking the initial conditions into account, they can be simplified to the following form:

(19) $$\rho \frac{\partial u}{\partial t}=-\frac{\partial p}{\partial x}+\frac{\partial \tau_{xy}}{\partial y},$$

(20) $$0=-\rho g-\frac{\partial p}{\partial y}.$$

From Equations (18) and (19), we can derive that

(21) $$\left(1+\lambda_{1}\frac{\partial }{\partial t}\right)\frac{\partial u}{\partial t}+\frac{1}{\rho }\left(1+\lambda_{1}\frac{\partial }{\partial t}\right)\frac{\partial p}{\partial x}=\nu \left(1+\lambda_{2}\frac{\partial }{\partial t}\right)\frac{\partial^{2}u}{\partial y^{2}}.$$

### 2.2. Dimensionless Analysis

Since this research focuses on an unsteady-flow problem, and the main flow is triggered by the horizontal oscillation of a flat plate, the appropriate time scale is the reciprocal of the externally applied oscillation frequency. Subsequently, the velocity scale is determined based on the balance between the inertial term and the viscous friction term. Correspondingly, by choosing *d* as the length scale, ν/d as the velocity scale, 1/ω as the time scale, and $\rho \nu^{2}/d^{2}$ as the pressure scale, the governing equations can be nondimensionalized [12]. The thickness *d* is a critical length parameter, which maps the flow domain from [−d,0] to [−1,0] through nondimensionalization, thus simplifying the expression of boundary conditions. The velocity scale ν/d is selected to balance viscous and inertial forces. The time scale is determined by oscillation frequency ω: Since the problem involves externally imposed periodic oscillations, the time scale naturally aligns with the oscillation period 1/ω. The choice of the characteristic scale of pressure is due to the fact that the pressure gradient balances viscous forces. Given that μ=ρν, the pressure scale becomes $\rho \nu^{2}/d^{2}$, ensuring consistency in the momentum equation. These selected characteristic scales give rise to a set of nondimensional parameters, and the specifically introduced nondimensional numbers are as follows:

(22) $$Oh=\frac{\nu }{d^{2}\omega },\;El=\frac{\lambda_{1}\nu }{d^{2}},\;\tilde{\lambda }=\frac{\lambda_{2}}{\lambda_{1}},\;De=\lambda_{1}\omega =\frac{El}{Oh},\;\chi =\frac{gd^{3}}{2\nu^{2}},\;Re=\frac{U_{0}d}{\nu },\;Ca=\frac{\rho \nu^{2}}{\sigma d}.$$

where the Ohnesorge number Oh reflects the relative magnitudes among the viscous force, the surface-tension force, and the inertial force; the Elastic number El is used to characterize the influence of the fluid’s elastic properties on the flow, representing the ratio of elastic stress to viscous stress and reflecting the intensity of the fluid’s elastic effect; $\tilde{\lambda }$ represents the ratio of the delay time to the relaxation time, reflecting the dynamic process by which the fluid’s internal structure returns to equilibrium; the Deborah number De is used to characterize the elastic effect of the fluid; the Galileo χ reflects the relative action between the gravitational force and the viscous force, characterizing the tendency of gravity-driven flow; the Reynolds number Re is used to characterize the influence of the oscillation amplitude; and the capillary number Ca is used to represent the influence of the surface-tension force, representing the ease of interface deformation.

After nondimensionalizing Equations (7)–(10), (20) and (21), the following results can be obtained:

(23) $$\frac{\partial U}{\partial t}+De\frac{\partial^{2}U}{\partial t^{2}}+Oh\frac{\partial P}{\partial x}+El\frac{\partial^{2}P}{\partial t\partial x}=Oh\frac{\partial^{2}U}{\partial y^{2}}+\tilde{\lambda }El\frac{\partial^{3}U}{\partial t\partial y^{2}},$$

(24) $$\frac{\partial P}{\partial y}=-2\chi ,$$

(25) U=Recost,aty=−1,

(26) $$El\frac{\partial^{2}U}{\partial t^{2}}=Oh^{2}\frac{\partial^{2}U}{\partial y^{2}}+\tilde{\lambda }ElOh\frac{\partial^{3}U}{\partial t\partial y^{2}},\;at\;y=0,$$

(27) P=0,aty=0.

### 2.3. Base Flow Solution

By the method of separation of variables, we obtain the unsteady flow solution on the oscillating plane:

(28) U(y,t)=Re−Resinh(By)eitsinhB,

(29) V(y,t)=0,

(30) P(y)=−2χy.

where the parameters $B=\sqrt{\frac{1}{Oh}i\frac{E}{F}}$, E=1+iDe and $F=1+\tilde{\lambda }Dei$. Here, Re[⋯] stands for the real part of the complex function within the brackets.

By performing nondimensionalization on Equations (14)–(16), the following nondimensionalized equations can be obtained:

(31) $$T_{xx}+De\frac{\partial T_{xx}}{\partial t}-2ElT_{xy}\frac{\partial U}{\partial y}=-2\tilde{\lambda }El{\left(\frac{\partial U}{\partial y}\right)}^{2},$$

(32) $$T_{yy}+De\frac{\partial T_{yy}}{\partial t}=0,$$

(33) $$T_{xy}+De\frac{\partial T_{xy}}{\partial t}=\frac{\partial U}{\partial y}+\tilde{\lambda }De\frac{\partial^{2}U}{\partial t\partial y}.$$

By solving the above system of equation, the following results can be obtained:

(34) $$T_{yy}\left(y,t\right)=0,$$

(35) Txy(y,t)=1E(∂U∂y+λ˜De∂2U∂t∂y),=Re−ReBFcosh(By)eitEsinhB,

(36) Txx(y,t)=2ElE(1E−λ˜)(∂U∂y)2+1EDe∂U∂y∂2U∂y∂t,=Re2ElRe2B2(coshBy)2(eit)2(1−λ˜)E2(sinhB)2.

Thus, the relevant solutions of the basic flow are obtained.

## 3. Orr–Sommerfeld Boundary Value Problem

To investigate the primary instability, we introduce a two-dimensional infinitesimal perturbation to the basic flow. Therefore, the basic flow velocity U(y,t) can be superimposed with a small perturbation $\tilde{u}\left(x,y,t\right)$; that is, the horizontal velocity component satisfies $u\left(x,y,t\right)=U\left(y,t\right)+\tilde{u}\left(x,y,t\right)$, where the magnitude of $\tilde{u}$ is much smaller than that of the basic flow velocity *U*. The same principle applies to other forms of perturbations. The form of this perturbation is as follows:

(37) $$\begin{array}{c} u=U+\tilde{u},\;v=\tilde{v},\;p=P+\tilde{p},\;h=\tilde{h},\\ \tau_{xx}=T_{xx}+{\tilde{\tau }}_{xx},\;\tau_{yy}={\tilde{\tau }}_{yy},\;\tau_{xy}=\tau_{yx}=T_{xy}+{\tilde{\tau }}_{xy}. \end{array}$$

Substituting the above-mentioned perturbation into the governing equations and boundary conditions in dimensionless form then linearizing the perturbation equations based on the basic flow, the following linearized perturbation equations can be obtained:

(38) $$\frac{\partial \tilde{u}}{\partial x}+\frac{\partial \tilde{v}}{\partial y}=0,$$

(39) $$\begin{array}{c} \frac{1}{Oh}\frac{\partial^{2}\tilde{u}}{\partial y\partial t}+U\frac{\partial^{2}\tilde{u}}{\partial y\partial x}+\tilde{v}\frac{\partial^{2}U}{\partial y^{2}}-\frac{1}{Oh}\frac{\partial^{2}\tilde{v}}{\partial x\partial t}-U\frac{\partial^{2}\tilde{v}}{\partial x^{2}}=\frac{\partial^{2}{\tilde{\tau }}_{xx}}{\partial y\partial x}+\frac{\partial^{2}{\tilde{\tau }}_{xy}}{\partial y^{2}}-\frac{\partial^{2}{\tilde{\tau }}_{xy}}{\partial x^{2}}-\frac{\partial^{2}{\tilde{\tau }}_{yy}}{\partial x\partial y}, \end{array}$$

(40) $$\begin{array}{c} {\tilde{\tau }}_{xx}+De\frac{\partial {\tilde{\tau }}_{xx}}{\partial t}+ElU\frac{\partial {\tilde{\tau }}_{xx}}{\partial x}+El\tilde{v}\frac{\partial T_{xx}}{\partial y}-2ElT_{xx}\frac{\partial \tilde{u}}{\partial x}-2ElT_{xy}\frac{\partial \tilde{u}}{\partial y}-2El{\tilde{\tau }}_{xy}\frac{\partial U}{\partial y}\\ =2\frac{\partial \tilde{u}}{\partial x}+\tilde{\lambda }\left(2De\frac{\partial \tilde{u}}{\partial x}+2ElU\frac{\partial^{2}\tilde{u}}{\partial x^{2}}-4El\frac{\partial U}{\partial y}\frac{\partial \tilde{u}}{\partial y}-2El\frac{\partial \tilde{v}}{\partial x}\frac{\partial U}{\partial y}\right), \end{array}$$

(41) $$\begin{array}{c} {\tilde{\tau }}_{yy}+De\frac{\partial {\tilde{\tau }}_{yy}}{\partial t}+ElU\frac{\partial {\tilde{\tau }}_{yy}}{\partial x}-2ElT_{xy}\frac{\partial \tilde{v}}{\partial x}=2\frac{\partial \tilde{v}}{\partial y}+\tilde{\lambda }\left(2De\frac{\partial^{2}\tilde{v}}{\partial t\partial y}+2ElU\frac{\partial^{2}\tilde{v}}{\partial x\partial y}\right.\\ \left.-2El\frac{\partial U}{\partial y}\frac{\partial \tilde{v}}{\partial x}\right), \end{array}$$

(42) $$\begin{array}{c} {\tilde{\tau }}_{xy}+De\frac{\partial {\tilde{\tau }}_{xy}}{\partial t}+ElU\frac{\partial {\tilde{\tau }}_{xy}}{\partial x}+El\tilde{v}\frac{\partial T_{xy}}{\partial y}-El{\tilde{\tau }}_{yy}\frac{\partial U}{\partial y}-ElT_{xx}\frac{\partial \tilde{v}}{\partial x}=\frac{\partial \tilde{u}}{\partial y}+\frac{\partial \tilde{v}}{\partial x}\\ +\tilde{\lambda }\left(De\frac{\partial^{2}\tilde{u}}{\partial t\partial y}+De\frac{\partial^{2}\tilde{v}}{\partial t\partial x}+ElU\frac{\partial^{2}\tilde{u}}{\partial x\partial y}+ElU\frac{\partial^{2}\tilde{v}}{\partial x^{2}}+El\tilde{v}\frac{\partial^{2}U}{\partial y^{2}}-2El\frac{\partial U}{\partial y}\frac{\partial \tilde{v}}{\partial y}\right). \end{array}$$

(43) $$\tilde{u}=0,\;\tilde{v}=0,\;at\;y=-1,$$

(44) $$-{\tilde{\tau }}_{xy}-\left(\frac{\partial^{3}U}{\partial y^{3}}+\tilde{\lambda }De\frac{\partial^{4}U}{\partial t\partial y^{3}}\right)\tilde{h}+\frac{\partial \tilde{h}}{\partial x}T_{xx}=0,\;at\;y=0,$$

(45) $$-2T_{xy}\frac{\partial \tilde{h}}{\partial x}+{\tilde{\tau }}_{yy}-\tilde{p}+2\chi \tilde{h}=\frac{1}{Ca}\frac{\partial^{2}\tilde{h}}{\partial x^{2}},\;at\;y=0,$$

(46) $$\frac{1}{Oh}\frac{\partial \tilde{h}}{\partial t}+U\frac{\partial \tilde{h}}{\partial x}=\tilde{v},\;at\;y=0.$$

Equation (39) is obtained by eliminating the pressure term after combining the momentum equations.

We assume that the normal mode solution [18] of the perturbation equation is as follows:

(47) $$\begin{array}{c} \tilde{u}=\hat{u}\left(y,t\right)e^{ikx},\;\tilde{v}=\hat{v}\left(y,t\right)e^{ikx},\;\tilde{h}=\hat{\eta }\left(t\right)e^{ikx},\\ {\tilde{\tau }}_{xx}={\hat{\tau }}_{xx}\;e^{ikx},\;{\tilde{\tau }}_{xy}={\tilde{\tau }}_{yx}={\hat{\tau }}_{xy}\;e^{ikx},\;{\tilde{\tau }}_{yy}={\hat{\tau }}_{yy}\;e^{ikx}. \end{array}$$

where *k* represents the wave number, and the symbol “^” is used to denote the amplitude of the perturbation variable.

By substituting the previously assumed normal mode solution of the perturbation into Equations (38)–(46) and conducting a series of derivations and calculations, a new, linearized equation composed of the perturbation amplitudes can be obtained. The specific equation is as follows:

(48) $$ik\hat{u}+\frac{\partial \hat{v}}{\partial y}=0,$$

(49) $$\frac{1}{Oh}\frac{\partial^{2}\hat{u}}{\partial y\partial t}+ikU\frac{\partial \hat{u}}{\partial y}+\hat{v}\frac{\partial^{2}U}{\partial y^{2}}-\frac{1}{Oh}ik\frac{\partial \hat{v}}{\partial t}+k^{2}U\hat{v}=ik\frac{\partial {\hat{\tau }}_{xx}}{\partial y}+\frac{\partial^{2}{\hat{\tau }}_{xy}}{\partial y^{2}}+k^{2}{\hat{\tau }}_{xy}-ik\frac{\partial {\hat{\tau }}_{yy}}{\partial y},$$

(50) $$\begin{array}{c} {\hat{\tau }}_{xx}+De\frac{\partial {\hat{\tau }}_{xx}}{\partial t}+ikElU{\hat{\tau }}_{xx}=2ik\hat{u}+\tilde{\lambda }\left(2ikDe\frac{\partial \hat{u}}{\partial t}-2k^{2}ElU\hat{u}-4El\frac{\partial U}{\partial y}\frac{\partial \hat{u}}{\partial y}-2ikEl\hat{v}\frac{\partial U}{\partial y}\right)\\ -El\hat{v}\frac{\partial T_{xx}}{\partial y}+2ikElT_{xx}\hat{u}+2ElT_{xy}\frac{\partial \hat{u}}{\partial y}+2El\frac{\partial U}{\partial y}{\hat{\tau }}_{xy}, \end{array}$$

(51) $${\hat{\tau }}_{yy}+De\frac{\partial {\hat{\tau }}_{yy}}{\partial t}+ikElU{\hat{\tau }}_{yy}-2ikElT_{xy}\hat{v}=2\frac{\partial \hat{v}}{\partial y}+\tilde{\lambda }\left(2De\frac{\partial^{2}\hat{v}}{\partial t\partial y}+2ikElU\frac{\partial \hat{v}}{\partial y}-2ikEl\frac{\partial U}{\partial y}\hat{v}\right),$$

(52) $$\begin{array}{c} {\hat{\tau }}_{xy}+De\frac{\partial {\hat{\tau }}_{xy}}{\partial t}+ikElU{\hat{\tau }}_{xy}=\frac{\partial \hat{u}}{\partial y}+ik\hat{v}+\tilde{\lambda }\left(De\frac{\partial^{2}\hat{u}}{\partial t\partial y}+ikDe\frac{\partial \hat{v}}{\partial t}+ikElU\frac{\partial \hat{u}}{\partial y}-k^{2}ElU\hat{v}\right.\\ \left.+El\hat{v}\frac{\partial^{2}U}{\partial y^{2}}-2El\frac{\partial U}{\partial y}\frac{\partial \hat{v}}{\partial y}\right)-El\hat{v}\frac{1}{E}\left(\frac{\partial^{2}U}{\partial y^{2}}+\tilde{\lambda }De\frac{\partial^{3}U}{\partial t\partial y^{2}}\right)+El\frac{\partial U}{\partial y}\frac{1}{E+ikElU}[2\frac{\partial \hat{v}}{\partial y}\\ +\tilde{\lambda }\left(2De\frac{\partial^{2}\hat{v}}{\partial t\partial y}+2ikElU\frac{\partial \hat{v}}{\partial y}-2ikEl\hat{v}\frac{\partial U}{\partial y}\right)+2\frac{El}{E}\left(\frac{\partial U}{\partial y}+\tilde{\lambda }De\frac{\partial^{2}U}{\partial t\partial y}\right)ik\hat{v}]+ikEl\hat{v}\frac{2El}{E}\\ \times \left[\left(\frac{1}{E}-\tilde{\lambda }\right){\left(\frac{\partial U}{\partial y}\right)}^{2}+\frac{1}{E}De\frac{\partial U}{\partial y}\frac{\partial^{2}U}{\partial y\partial t}\right]. \end{array}$$

The relevant boundary conditions are as follows:

(53) $$\hat{u}=0,\;\hat{v}=0,\;at\;\;y=-1,$$

(54) $$-{\hat{\tau }}_{xy}-\left(\frac{\partial^{3}U}{\partial y^{3}}+\tilde{\lambda }De\frac{\partial^{4}U}{\partial t\partial y^{3}}\right)\hat{\eta }+ik\hat{\eta }T_{xx}=0,\;at\;y=0,$$

(55) $$-2T_{xy}k^{2}\hat{\eta }+ik{\hat{\tau }}_{yy}+\frac{1}{Oh}\frac{\partial \hat{u}}{\partial t}+ikU\hat{u}+\hat{v}\frac{\partial U}{\partial y}-ik{\hat{\tau }}_{xx}-\frac{\partial {\hat{\tau }}_{xy}}{\partial y}+2\chi ik\hat{\eta }=-\frac{1}{Ca}ik^{3}\hat{\eta },\;at\;y=0,$$

(56) $$\frac{1}{Oh}\frac{\partial \hat{\eta }}{\partial t}+ikU\hat{\eta }=\hat{v},\;at\;y=0.$$

Based on the following specific relationship existing between the velocity components *u* and *v* and the stream function:

(57) $$\tilde{u}=\partial_{y}\tilde{\psi }\;,\;\tilde{v}=-\partial_{x}\tilde{\psi }.$$

We introduce a stream function in the following form:

(58) $$\tilde{\psi }\left(x,y,t\right)=\hat{\phi }\left(y,t\right)e^{ikx}.$$

Then, the relationship between the perturbation amplitude and the stream function amplitude can be obtained:

(59) $$\hat{u}=\partial_{y}\hat{\phi }\;,\;\hat{v}=-ik\hat{\phi }.$$

Next, by substituting Equation (59) into the linearized Equations (48)–(56), the corresponding Orr–Sommerfeld boundary value problem (O-S BVP) for the viscoelastic fluid studied in this paper can be obtained:

(60) $$\frac{1}{Oh}\frac{\partial^{3}\hat{\phi }}{\partial y^{2}\partial t}+ikU\frac{\partial^{2}\hat{\phi }}{\partial y^{2}}-ik\hat{\phi }\frac{\partial^{2}U}{\partial y^{2}}-\frac{1}{Oh}k^{2}\frac{\partial \hat{\phi }}{\partial t}-ik^{3}U\hat{\phi }=ik\frac{\partial {\hat{\tau }}_{xx}}{\partial y}+\frac{\partial^{2}{\hat{\tau }}_{xy}}{\partial y^{2}}+k^{2}{\hat{\tau }}_{xy}-ik\frac{\partial {\hat{\tau }}_{yy}}{\partial y},$$

(61) $$\hat{\phi }=\partial_{y}\hat{\phi }=0,\;at\;y=-1,$$

(62) $$-{\hat{\tau }}_{xy}-\left(\frac{\partial^{3}U}{\partial y^{3}}+\tilde{\lambda }De\frac{\partial^{4}U}{\partial t\partial y^{3}}\right)\hat{\eta }+ik\hat{\eta }T_{xx}=0,\;at\;y=0,$$

(63) $$-2T_{xy}k^{2}\hat{\eta }+ik{\hat{\tau }}_{yy}+\frac{1}{Oh}\frac{\partial^{2}\hat{\phi }}{\partial y\partial t}+ikU\frac{\partial \hat{\phi }}{\partial y}-ik\hat{\phi }\frac{\partial U}{\partial y}-ik{\hat{\tau }}_{xx}-\frac{\partial {\hat{\tau }}_{xy}}{\partial y}+2\chi ik\hat{\eta }=-\frac{1}{Ca}ik^{3}\hat{\eta },\;at\;y=0,$$

(64) $$\frac{1}{Oh}\frac{\partial \hat{\eta }}{\partial t}+ikU\hat{\eta }=-ik\hat{\phi },\;at\;y=0.$$

where

(65) $${\hat{\tau }}_{yy}=\frac{1}{E+ikElU}\left[2\frac{\partial \hat{v}}{\partial y}+\tilde{\lambda }\left(2De\frac{\partial^{2}\hat{v}}{\partial t\partial y}+2ikElU\frac{\partial \hat{v}}{\partial y}-2ikEl\frac{\partial U}{\partial y}\hat{v}\right)+2ik\frac{El}{E}\left(\frac{\partial U}{\partial y}+\tilde{\lambda }De\frac{\partial^{2}U}{\partial t\partial y}\right)\hat{v}\right],$$

(66) $$\begin{array}{cc} {\hat{\tau }}_{xy} & =\frac{1}{E+ikElU}\{\frac{\partial \hat{u}}{\partial y}+ik\hat{v}+\hat{\lambda }\left(De\frac{\partial^{2}\hat{u}}{\partial t\partial y}+ikDe\frac{\partial \hat{v}}{\partial t}+ikElU\frac{\partial \hat{u}}{\partial y}-k^{2}ElU\hat{v}+El\hat{v}\frac{\partial^{2}U}{\partial y^{2}}-2El\frac{\partial U}{\partial y}\frac{\partial \hat{v}}{\partial y}\right)\\ & -\frac{El}{E}\hat{v}\left(\frac{\partial^{2}U}{\partial y^{2}}+\hat{\lambda }De\frac{\partial^{3}U}{\partial t\partial y^{2}}\right)+2ik\hat{v}\frac{El^{2}}{E}\left[\left(\frac{1}{E}-\hat{\lambda }\right){\left(\frac{\partial U}{\partial y}\right)}^{2}+\frac{1}{E}De\frac{\partial U}{\partial y}\frac{\partial^{2}U}{\partial y\partial t}\right]+\frac{\partial U}{\partial y}\frac{El}{E+ikElU}\\ & \times \left[2\frac{\partial \hat{v}}{\partial y}+\tilde{\lambda }\left(2De\frac{\partial^{2}\hat{v}}{\partial t\partial y}+2ikElU\frac{\partial \hat{v}}{\partial y}-2ikEl\hat{v}\frac{\partial U}{\partial y}\right)+2ik\frac{El}{E}\left(\frac{\partial U}{\partial y}+\tilde{\lambda }De\frac{\partial^{2}U}{\partial t\partial y}\right)\hat{v}\right]\}, \end{array}$$

(67) $$\begin{array}{cc} {\hat{\tau }}_{xx} & =\frac{1}{E+ikElU}\{2ik\hat{u}+\tilde{\lambda }\left(2ikDe\frac{\partial \hat{u}}{\partial t}-2ElUk^{2}\hat{u}-4El\frac{\partial U}{\partial y}\frac{\partial \hat{u}}{\partial y}-2ikEl\hat{v}\frac{\partial U}{\partial y}\right)-2\frac{El^{2}}{E}\hat{v}[2\left(\frac{1}{E}-\tilde{\lambda }\right)\\ & \times \frac{\partial U}{\partial y}\frac{\partial^{2}U}{\partial y^{2}}+\frac{1}{E}De\frac{\partial^{2}U}{\partial y^{2}}\frac{\partial^{2}U}{\partial y\partial t}+\frac{1}{E}De\frac{\partial U}{\partial y}\frac{\partial^{3}U}{\partial y^{2}\partial t}]+4ik\frac{El^{2}}{E}\hat{u}\left[\left(\frac{1}{E}-\tilde{\lambda }\right){\left(\frac{\partial U}{\partial y}\right)}^{2}+\frac{1}{E}De\frac{\partial U}{\partial y}\frac{\partial^{2}U}{\partial y\partial t}\right]\\ & +2\frac{El}{E}\frac{\partial \hat{u}}{\partial y}\left(\frac{\partial U}{\partial y}+\tilde{\lambda }De\frac{\partial^{2}U}{\partial t\partial y}\right)+\frac{2El}{E+ikElU}\frac{\partial U}{\partial y}\{\frac{\partial \hat{u}}{\partial y}+ik\hat{v}+\tilde{\lambda }\left(De\frac{\partial^{2}\hat{u}}{\partial t\partial y}+ikDe\frac{\partial \hat{v}}{\partial t}+ikElU\frac{\partial \hat{u}}{\partial y}\right.\\ & -k^{2}ElU\hat{v}+El\hat{v}\frac{\partial^{2}U}{\partial y^{2}}-2El\frac{\partial U}{\partial y}\frac{\partial \hat{v}}{\partial y})-\frac{El}{E}\hat{v}\left(\frac{\partial^{2}U}{\partial y^{2}}+\tilde{\lambda }De\frac{\partial^{3}U}{\partial t\partial y^{2}}\right)+2ik\hat{v}\frac{El^{2}}{E}\left[\left(\frac{1}{E}-\tilde{\lambda }\right){\left(\frac{\partial U}{\partial y}\right)}^{2}\right.\\ & +\frac{1}{E}De\frac{\partial U}{\partial y}\frac{\partial^{2}U}{\partial y\partial t}]+\frac{\partial U}{\partial y}\frac{El}{E+ikElU}[2\frac{\partial \hat{v}}{\partial y}+\tilde{\lambda }\left(2De\frac{\partial^{2}\hat{v}}{\partial t\partial y}+2ikElU\frac{\partial \hat{v}}{\partial y}-2ikEl\hat{v}\frac{\partial U}{\partial y}\right)\\ \; & +2ik\frac{El}{E}\left(\frac{\partial U}{\partial y}+\tilde{\lambda }De\frac{\partial^{2}U}{\partial t\partial y}\right)\hat{v}]\}\}. \end{array}$$

This Orr–Sommerfeld boundary-value problem (O-S BVP) forms a Floquet system. When dealing with finite-wavelength instability, the Floquet system needs to be solved numerically, while for long-wavelength instability, we can obtain an analytical solution by expanding in series of *k*. Next, we will explore the long-wavelength solution.

## 4. Long-Wavelength Expansion

For the time-dependent Orr–Sommerfeld boundary-value problem (O-S BVP), based on Floquet theory, we carry out a long-wave expansion and assume its solution to be in the following Floquet form:

(68) $$\left[\begin{array}{c} \hat{\phi }\left(y,t\right)\\ \hat{\eta }\left(t\right) \end{array}\right]=e^{\delta t}\left[\begin{array}{c} \phi_{0}\left(y,t\right)+k\phi_{1}\left(y,t\right)+\ldots \\ \eta_{0}\left(t\right)+k\eta_{1}\left(y,t\right)+\ldots \end{array}\right].$$

The Floquet exponent takes the following form:

(69) $$\delta =\delta_{0}+k\delta_{1}+k^{2}\delta_{2}+\cdots .$$

To comprehensively consider the influence of surface tension in the first-order formula, we introduce the capillary number $Ca∼O(k^{2})$. By substituting Equations (68) and (69) into the Orr–Sommerfeld boundary-value problem (O-S BVP) and extracting the leading-order $O(k^{0})$ terms, the following system of equations is obtained:

(70) $$\left(\frac{1}{Oh}D^{2}-\frac{1}{E}\tilde{\lambda }DeD^{4}\right)\left(\partial_{t}\phi_{0}+\delta_{0}\phi_{0}\right)=\frac{1}{E}D^{4}\phi_{0},$$

(71) $$\phi_{0}=D\phi_{0}=0,\;at\;y=-1,$$

(72) $$\frac{1}{E}D^{2}\phi_{0}+\frac{1}{E}\tilde{\lambda }DeD^{2}\left(\partial_{t}\phi_{0}+\delta_{0}\phi_{0}\right)+\left(\frac{\partial^{3}U}{\partial y^{3}}+\tilde{\lambda }De\frac{\partial^{4}U}{\partial t\partial y^{3}}\right)\eta_{0}=0,\;at\;y=-1,$$

(73) $$\left(\frac{1}{Oh}D-\frac{1}{E}\tilde{\lambda }DeD^{3}\right)\left(\partial t\phi_{0}+\delta_{0}\phi_{0}\right)=\frac{1}{E}D^{3}\phi_{0},\;at\;y=0,$$

(74) $$\frac{1}{Oh}\left(\partial_{t}\eta_{0}+\delta_{0}\eta_{0}\right)=0,\;at\;y=0.$$

where $D=\frac{\partial }{\partial y}$ is a differential operator. Through the analysis and derivation of (74), it can be concluded that $\delta_{0}=0$. Given that $\eta_{0}\left(t\right)$ is a periodic function of *t*, the kinematic boundary condition (74) will inevitably yield a feasible solution $\delta_{0}=0$. On this basis, we select $\eta_{0}=1$. Such a choice is general and will not affect the universality of the conclusion. Because once $\delta_{0}\neq 0$, the damped Floquet mode pointed out by Yih [7] in 1968 will emerge, within the scope of the current research, our focus is not on the damped mode.

Upon solving the above system of equations, the following leading-order solution can be obtained:

(75) ϕ0(y,t)=Re−ReBE1−coshB(y+1)eitsinhBcoshB.

Next, through systematically collecting and collating the first-order O(k) terms, we obtain the following system of equations:

(76) $$D^{4}\phi_{1}=iE\left(UD^{2}\phi_{0}-\phi_{0}D^{2}U\right)-i\tilde{\lambda }El\left(UD^{4}\phi_{0}-\phi_{0}D^{4}U\right)-i\frac{El}{E}\phi_{0}D^{4}U,$$

(77) $$\phi_{1}=D\phi_{1}=0,\;at\;y=-1,$$

(78) $$\begin{array}{c} D^{2}\phi_{1}=-i\tilde{\lambda }El\left(UD^{2}\phi_{0}-\phi_{0}D^{2}U+2DUD\phi_{0}\right)-i\frac{El}{E}\left(\phi_{0}D^{2}U-2DUD\phi_{0}\right)-ED^{3}U\eta_{1}\\ +2iEl\left(\frac{1}{E}-\tilde{\lambda }\right){\left(DU\right)}^{2}\eta_{0},\;at\;y=0, \end{array}$$

(79) $$\begin{array}{c} D^{3}\phi_{1}=iE\left(UD\phi_{0}-\phi_{0}DU\right)+i\tilde{\lambda }El\left(DUD^{2}\phi_{0}-UD^{3}\phi_{0}-D\phi_{0}D^{2}U+\phi_{0}D^{3}U\right)\\ -iElD^{2}\phi_{0}DU+i\frac{El}{E}\left(D^{2}UD\phi_{0}-\phi_{0}D^{3}U\right)+2iE\left(\chi +\frac{k^{2}}{2Ca}\right)\eta_{0},\;at\;y=0, \end{array}$$

(80) $$\frac{1}{Oh}\left(\partial_{t}\eta_{1}+\delta_{1}\eta_{0}+\delta_{0}\eta_{1}\right)=-i\phi_{0}-iU\eta_{0},\;at\;y=0.$$

Given that $\phi_{0}\left(y,t\right)$, U(y,t), and $\eta_{0}\left(t\right)$ are all time-periodic functions, for $\eta_{1}\left(t\right)$ to be a periodic solution derived from the kinematic boundary conditions, it is necessary that $\delta_{1}=0$. As a result, Equation (80) can be reduced to the following form:

(81) $$\frac{1}{Oh}\partial_{t}\eta_{1}=-i\left(\phi_{0}+U\right).$$

the periodic solution of $\eta_{1}$ can be obtained:

(82) η1=−iOhReImBE(1−coshB)sinhBcoshBeit.

Here, Im[⋯] represents the imaginary part of this complex function.

The first-order equation is composed of a steady-state part and an unsteady-state part. In view of the fact that the Floquet exponent has no relation with time, only by obtaining the first-order steady-state solution can we smoothly derive the next-order Floquet exponent $\delta_{2}$ and the next-order solution. Based on this, we neglect the unsteady-state part and then obtain the time-independent first-order steady-state system of equations. By carrying out the solution to this first-order steady-state system of equations, we finally obtain the following:

(83) ϕ1s(y)=ϕ1c(y)−iRe2Re[L0]+iRe2Re[L1]−iRe2Re[L2]+iRe2Re[L3].

where superscript “S” denotes the steady solution and the expressions corresponding to each parameter are as follows:

(84) $$\phi_{1c}\left(y\right)=A_{0}+A_{1}y+A_{2}y^{2}+A_{3}y^{3},$$

(85) $$L_{0}=\frac{E^{2}\sinh By}{B\sinh^{2}B\cosh B},$$

(86) $$L_{1}=\frac{\tilde{\lambda }ElBE\sinh By}{\sinh^{2}B\cosh B},$$

(87) $$L_{2}=\frac{ElB\sinh By}{\sinh^{2}B\cosh B},$$

(88) $$L_{3}=\frac{ElB}{\sinh^{2}B\cosh B}\frac{\sinh[2B(2y+1)]}{32}-\frac{ElB^{5}}{\sinh^{2}B\cosh B}\frac{\sinh B}{48}y^{4},$$

(89) $$A_{0}=-2d_{1}+iRe^{2}\left(I_{0}+I_{1}-3I_{2}-2I_{3}\right),$$

(90) $$A_{1}=-3d_{1}+iRe^{2}\left(I_{1}-2I_{2}-3I_{3}\right),$$

(91) $$A_{2}=iRe^{2}I_{2},$$

(92) $$A_{3}=iRe^{2}I_{3}+d_{1},$$

(93) $$d_{1}=\frac{1}{3}iE\left(\chi +\frac{k^{2}}{2Ca}\right).$$

where

(94) I3=Re−16B4E2(1−coshB)+2λ˜ElB4EcoshB−74ElB4coshB−B2E2sinh2BcoshB,

(95) I2=Re122λ˜ElB3EsinhB−2ElB3sinhB−OhB4E2i(1−coshB)+2ElB2(1E−λ˜)coshB−1BElB2coshBsinh2BcoshB,

(96) I1=ReE2coshB−λ˜ElB2EcoshB+ElB2coshB−116ElB2coshB−112ElB5coshBsinh2BcoshB,

(97) I0=Re−1BE2sinhB+λ˜ElBEsinhB−ElBsinhB+132ElBsinhB+148ElB5sinhBsinh2BcoshB.

In this paper, in order to accurately calculate the Floquet exponent $\delta_{2}$, under the consideration of the second-order $O(k^{2})$, analyze and research the approximate value of the kinematic boundary condition when y=0:

(98) $$\frac{1}{Oh}\left(\partial_{t}\eta_{2}+\delta_{2}\eta_{0}+\delta_{1}\eta_{1}+\delta_{0}\eta_{2}\right)=-i\phi_{1}-iU\eta_{1}.$$

$\delta_{2}$ is independent of time, and its expression is given by the first-order solution.

(99) $$\begin{array}{cc} \delta_{2} & =-Ohi\left(\phi_{1}^{\left(s\right)}+{\left(U\eta_{1}\right)}^{\left(s\right)}\right)\\ \; & =Oh\left[2id_{1}+Re^{2}\left(I_{0}+I_{1}+I_{4}-3I_{2}-2I_{3}\right)\right]. \end{array}$$

where

(100) I4=ReElB32sinhBcoshB.

Based on $\delta_{0}$, $\delta_{1}$, and $\delta_{2}$, the expression of the Floquet exponent δ can be given:

(101) $$\begin{array}{cc} \delta & =k^{2}\delta_{2}+O\left(k^{3}\right),\\ & =k^{2}Oh\left[2id_{1}+Re^{2}\left(I_{0}+I_{1}+I_{4}-3I_{2}-2I_{3}\right)\right]+O\left(k^{3}\right),\\ & =k^{2}Oh\left[\underset{VECT}{\underset{︸}{Re^{2}\left(I_{0}+I_{1}+I_{4}-3I_{2}-2I_{3}\right)}}-\underset{GT}{\underset{︸}{\frac{2E\chi }{3}}}-\underset{ST}{\underset{︸}{\frac{Ek^{2}}{3Ca}}}\right]+O\left(k^{3}\right). \end{array}$$

VECT, GT, and ST stand for the viscoelastic coupling term, the gravity term, and the surface-tension term, respectively. Equation (101) indicates that the Floquet exponent δ is just a straightforward combination of these three terms: VECT, GT, and ST. Additionally, when both gravity and surface tension are present, the value of the Floquet exponent δ will decline. To put it another way, gravity and surface tension contribute to the stabilization of long-wave modes.

Previously, we have determined the leading-order and first-order solutions of the kinematic boundary. As we found before, $\delta_{0}=0$ and $\delta_{1}=0$. In the long-wave limit where k→0, when $\delta_{2}>0$ or $\delta_{2}<0$, which is equivalent to meeting the following criteria, the amplitude of the infinitesimal perturbation $\left[\propto e^{\delta t}=e^{k^{2}\delta_{2}+O\left(k^{3}\right)}\right]$ will experience exponential growth or decay as time progresses:

(102) $$\frac{2E\chi }{Re^{2}}\;<\;L\;or\;\frac{2E\chi }{Re^{2}}\;>\;L.$$

where

(103) $$L=3\left(I_{0}+I_{1}+I_{4}-3I_{2}-2I_{3}\right)-\frac{Ek^{2}}{CaRe^{2}}.$$

Therefore, we can draw the conclusion that when $\delta_{2}<0$, long-wave perturbations are in a stable state; conversely, if $\delta_{2}>0$, long-wave perturbations are unstable.

To analyze the impact of surface tension on the long-wave mode separately, we introduce a new parameter, $\zeta^{*}=\frac{k^{2}}{Ca}$, which serves to represent the influence of surface tension. According to the neutral stability criterion $\delta_{2}=0$, the following relationship can be deduced:

(104) $$Re={\left[\frac{2E\chi +E\zeta^{*}}{3(I_{0}+I_{1}+I_{4}-3I_{2}-2I_{3})}\right]}^{\frac{1}{2}}.$$

According to the above-mentioned relationship, we can plot the neutral curve and then conduct a stability analysis.

**Figure 2:**
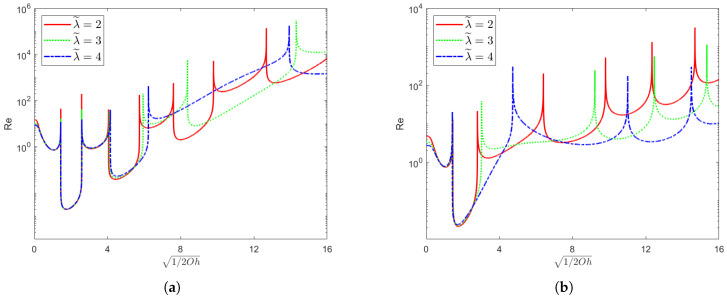
When χ=1 and $\zeta^{*}=0$, this figure depicts the neutral curves corresponding to different values of $\tilde{\lambda }$ in the $\left(\sqrt{1/2Oh},Re\right)$ plane. In (**a**), the elasticity number El=0.001; in (**b**), the elasticity number El=0.01.

Figure 2 demonstrates the variation in the Reynolds number Re with $\sqrt{1/2Oh}$ under the condition of k→0. Here, χ=1 and $\zeta^{*}=0$ are fixed, while the values of the elastic parameter El and the parameter $\tilde{\lambda }$ are altered. In the figure, the U-shaped neutral curves emerge within the bandwidth associated with the Ohnesorge number Oh. Evidently, long-wavelength instabilities are only present within these specific bandwidths. Beyond these bandwidths, all infinitesimal perturbations remain stable. Moreover, within each unstable bandwidth, there exists a critical Reynolds number; in other words, it corresponds to a critical amplitude of horizontal oscillation. Once this critical amplitude is exceeded, the long-wave mode becomes unstable. Notably, as the value of the elastic parameter El increases, the number of unstable bandwidths gradually decreases. Similarly, as $\tilde{\lambda }$ increases, the number of unstable bandwidths also decreases. However, in different unstable bandwidths, the intensity of the dominant unstable mode increases with the growth of the elastic parameter value and $\tilde{\lambda }$. The above results show that an increase in El and $\tilde{\lambda }$ stabilizes the system. These narrow spikes reflect the response of the liquid film to disturbances in the system at a specific $\sqrt{1/2Oh}$. Specifically, when the parameter $\sqrt{1/2Oh}$ related to the vibration frequency approaches certain critical values, the coupling between the elastic effects of the fluid (El and $\tilde{\lambda }$) and the external oscillation leads to drastic changes in the stability conditions. At this point, a higher Reynolds number Re is required for the system to reach a neutrally stable state. The appearance of these spikes indicates that the liquid film is extremely sensitive to disturbances near these specific frequencies, and minor parameter changes can cause a switch in the stability state.

**Figure 3:**
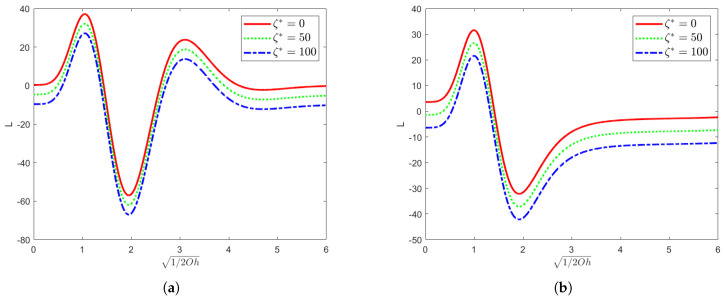
When the dimensionless parameter χ=1 and the Reynolds number Re=10, this figure shows the neutral curves for different values of $\zeta^{*}$ in the $\left(\sqrt{1/2Oh},L\right)$ plane. In (**a**), the elasticity number El=0.001; in (**b**), the elasticity number El=0.01.

Figure 3 shows the variation in the long-wave mode growth rate *L* with $\sqrt{1/2Oh}$ for a fixed dimensionless parameter χ=1 and the Reynolds number Re=10 with varying values of the elasticity parameter El and of the parameter indicating the effect of surface tension $\zeta^{*}$. In the O(k) approximation of the long-wave analysis, the maximum value of the long-wave mode growth rate *L* decreases with the increase in the value of $\zeta^{*}$ when the effect of surface tension is taken into account, indicating that the surface tension reduces the amplitude of the growth rate by suppressing the interfacial deformation and hindering the development of the long-wave perturbation. Moreover, the maximum value of *L* decreases when El increases, and its corresponding value of $\sqrt{1/2Oh}$ also decreases, indicating that the system is destabilized at lower $\sqrt{1/2Oh}$ (i.e., higher Oh) when El increases, which enlarges the stability region of the system. An increase in El indicates an increase in the elastic effect, and the elastic stress dissipates the perturbation energy, suppressing the growth of the long-wave modes and stabilizing the system. Mathematically, *L* quantifies the growth of long-wave modes over time under specific conditions. This clearly shows that surface tension plays a stabilizing role in the long wave modes in this case. This is because the decrease in the growth rate means that it is difficult for the long wave modes to develop and amplify, thus making the whole system more stable.

**Figure 4:**
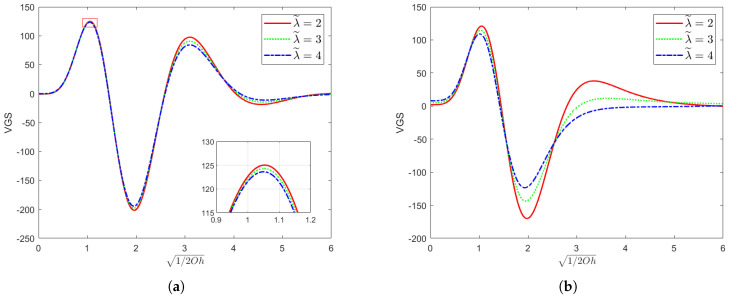
When the parameter χ=1, the Reynolds number Re=10, and $\zeta^{*}=1$, this figure presents the neutral curves corresponding to different values of $\tilde{\lambda }$ in the $\left(\sqrt{1/2Oh},VGS\right)$ plane, where VGS=VECT+GT+ST. In (**a**), the elasticity number El=0.001; in (**b**), the elasticity number El=0.01.

Figure 4 shows the variation in VGS with $\sqrt{1/2Oh}$ for a fixed dimensionless parameter χ=1, the Reynolds number Re=10, and parameter $\zeta^{*}=1$ with varying values of the elasticity parameter El and the parameter $\tilde{\lambda }$. From the figure, it can be clearly seen that as El and $\tilde{\lambda }$ keep increasing, the maximum value of VGS decreases gradually, and the decrease in the maximum value of VGS corresponds to the decrease in the Floquet index, which fully indicates that the system is tending to a stable state. Since $\tilde{\lambda }$ denotes the ratio of the delay time to the relaxation time, an increase in its value implies a change in the relative process by which the internal structure of the fluid returns to equilibrium after being subjected to an external force. Specifically, the delay time becomes longer than the relaxation time. This phenomenon indicates that when the fluid is perturbed, it has more time to adjust its internal structure to resist further deformation. At this point, the behavior of the fluid is more similar to that of an elastic solid, and to a certain extent, it can resist external perturbations more effectively, thus contributing to the stabilizing trend of the system.

## 5. Conclusions

This paper provides an insight into the linear stability analysis of the liquid film of Oldroyd-B fluid on an oscillating plate. By applying small perturbations to the elementary flow, the time-dependent Orr–Sommerfeld equation is successfully derived, and the Floquet system is constructed. In addition, an in-depth study using the method of series expansion is presented. In the long-wave region, the analytical solution of the time-dependent Orr–Sommerfeld equation is given with the help of Floquet theory and the assumption of the regular mode solution. By plotting the neutral curves and analyzing them in detail, it is found that the long-wave instability occurs only within a specific bandwidth associated with the Ohnesorge number Oh. As the elasticity parameter El and the parameter $\tilde{\lambda }$ gradually increase, the number of unstable bandwidths decreases, and the system tends toward a steady state. However, within different instability bandwidths, the intensity of the main unstable modes increases continuously with the increase in El and $\tilde{\lambda }$. Furthermore, in each instability bandwidth, the long-wave modes are unstable only when the associated Reynolds number exceeds a critical value or, equivalently, when the amplitude of the horizontal oscillations exceeds a critical value.

In the O(k) approximation for long-wave analysis, the parameter $\zeta^{*}=k^{2}/Ca$ is specifically introduced to characterize the effect of surface tension. It is shown that the maximum value of the long-wave mode growth rate *L* decreases with the increase in $\zeta^{*}$. This phenomenon clearly indicates that the surface tension plays a stabilizing role on the long-wave modes, making it difficult for them to develop and amplify, thus greatly enhancing the stability of the whole system. When the dimensionless parameters χ, the Reynolds number Re, and $\zeta^{*}$ are fixed, the maximum value of VGS decreases as the parameters El and $\tilde{\lambda }$ keep increasing. This change corresponds to a decrease in the Floquet index, which means that the system is approaching a steady state. Since $\tilde{\lambda }$ represents the ratio of the delay time to the relaxation time, the increase in its value means that the relative process of the fluid’s internal structure returning to the equilibrium state after being subjected to an external force changes, and the delay time becomes longer, which contributes to the system’s tendency to stabilize.

In summary, this paper comprehensively and clearly elucidates the influence mechanism of elastic parameters, the ratio of delay time and relaxation time, surface tension, and other factors on the stability of long-wave modes, which provides an important reference for related engineering applications and further theoretical research.

## Data Availability

The data that support the findings of this study are available from the corresponding author upon reasonable request.
